# Regulated Cell Death in Endometriosis

**DOI:** 10.3390/biom14020142

**Published:** 2024-01-23

**Authors:** Erqing Huang, Xiaoli Wang, Lijuan Chen

**Affiliations:** Department of Obstetrics and Gynecology, Union Hospital, Tongji Medical College, Huazhong University of Science and Technology, Wuhan 430022, China; m202176090@hust.edu.cn (E.H.);

**Keywords:** endometriosis, regulated cell death (RCD), apoptosis, pyroptosis, ferroptosis, cuproptosis, autophagy, target therapy

## Abstract

Regulated cell death (RCD) represents a distinct mode of cell demise, differing from accidental cell death (ACD), characterized by specific signaling cascades orchestrated by diverse biomolecules. The regular process of cell death plays a crucial role in upholding internal homeostasis, acting as a safeguard against biological or chemical damage. Nonetheless, specific programmed cell deaths have the potential to activate an immune–inflammatory response, potentially contributing to diseases by enlisting immune cells and releasing pro-inflammatory factors. Endometriosis, a prevalent gynecological ailment, remains incompletely understood despite substantial progress in unraveling associated signaling pathways. Its complexity is intricately tied to the dysregulation of inflammatory immune responses, with various RCD processes such as apoptosis, autophagic cell death, pyroptosis, and ferroptosis implicated in its development. Notably, limited research explores the association between endometriosis and specific RCD pathways like pyroptosis and cuproptosis. The exploration of regulated cell death in the context of endometriosis holds tremendous potential for further advancements. This article thoroughly reviews the molecular mechanisms governed by regulated cell death and their implications for endometriosis. A comprehensive understanding of the regulated cell death mechanism in endometriosis has the potential to catalyze the development of promising therapeutic strategies and chart the course for future research directions in the field.

## 1. Introduction

Endometriosis is defined as the appearance of endometrium-like tissue in areas other than the uterine cavity. Statistics indicate that approximately 10% of women in the reproductive age group globally experience this medical condition [1,2,3]. The primary manifestations of endometriosis encompass dysmenorrhea, chronic pelvic pain, infertility, dyspareunia, and potential dysfunction of the corresponding systems if lesions accumulate in the bladder and rectum. Presently, surgical resection of lesions remains the established gold standard for endometriosis treatment. However, the condition is prone to postoperative recurrence, and the management of the disease in the long term poses significant challenges. Estrogen-driven inflammation and immune dysregulation are recognized as pivotal components in the pathogenesis of endometriosis [4]. Substantial advancements have been achieved in elucidating the mechanisms underlying the inflammatory immune response in endometriosis in recent years. Certain studies propose a close association between the abnormal recruitment of immune cells, overactivation of pro-inflammatory factors, and processes such as oxidative stress, autophagy, and apoptosis in the context of endometriosis [5].

Two primary modes of cell death exist, namely accidental cell death (ACD) and regulated cell death (RCD), with ACD being unregulated and susceptible to damage from various physicochemical and biological factors. Subsequently, in 1972, the inaugural form of RCD, known as apoptosis, was discovered [6]. Through the study of apoptosis, it has been found that cell death can be genetically regulated, and even the pathogenesis of some tumors, heart disease, chronic kidney disease, and autoimmune diseases is related to anti-apoptosis [7,8,9]. This has led us to realize that cell death is not only a physiological process but can also lead to pathological processes. The RCD cascade reaction includes recognition, triggering, execution, and other effector molecules, leading to unique morphological, biochemical, and immunological features [10]. RCD is further classified into apoptotic and non-apoptotic subcategories [11]. Cells with an apoptotic RCD retain their membrane integrity and exhibit shrinkage of the cytoplasm, condensation of the chromatin, fragmentation of the nucleus, and blebbing of the plasma membrane [12]. Other non-apoptotic RCDs have also been discovered in recent years, including apoptosis, necroptosis, pyroptosis, ferroptosis, anoikis, cuproptosis, and so on. Many studies on endometriosis and RCD, such as apoptosis, ferroptosis, and pyroptosis, have occurred [13,14,15,16,17,18,19,20].

Do these RCD processes intricately interact with the pathophysiology of endometriosis? Could pivotal pathways or molecules emerging from RCD be deemed prospective targets for the therapeutic intervention of endometriosis? These inquiries warrant methodical consolidation and exploration in this comprehensive review. Herein, we present an exhaustive summary of the most recent literature delineating RCD pathways or regulators associated with endometriosis. Additionally, we will deliberate on potential RCD biomarkers and explore RCD-based therapeutic strategies for addressing endometriosis. Finally, we will expound on the prospective directions for future research on RCD mechanisms in the context of endometriosis.

## 2. Endometriosis Pathophysiology

In 1920, the predominant theory explaining the pathogenesis of endometriosis, known as “menstrual reflux,” was postulated [21]. The originator of this theory, Sampson, posited that fragments of endometrial tissue, carried by menstrual blood, traverse the fallopian tubes and enter the abdominal cavity. The ectopic endometrium can migrate and invade and is firmly implanted in the pelvis through vascular and neurogenesis. Ectopic endometrium can also change with the menstrual cycle, causing a chronic inflammatory response in the affected area, which is why patients with endometriosis are often found to have severe pelvic adhesions and chronic pelvic inflammation during surgical procedures. In fact, menstrual reflux is also prevalent in healthy women. This makes one wonder why some people are normal and others have endometriosis. Some studies have shown that the immune system’s failure to clear the ectopic endometrium in time or its failure to clear it is also one of the important causes of endometriosis [22,23]. It has been found that the expression of the anti-apoptotic genes is up-regulated in the eutopic and ectopic endometrium of patients with endometriosis, which proves that there is an anti-apoptotic phenomenon in endometriosis [24]. In addition to this, the proliferative capacity of the endometrium is significantly increased in patients with endometriosis [25,26]. These all contribute to the growth and adhesion of endometriosis lesions.

Endometriosis is defined as a hormone-dependent disorder, specifically a dysregulation of estrogen and progesterone [27,28]. In the eutopic and ectopic endometrium of endometriosis patients, progesterone resistance is detected, and progesterone receptors are reduced. The logic behind the use of progesterone for endometriosis comes from the positive effects that researchers have observed on endometriosis during pregnancy [29]. There are two types of progesterone receptors, PRA and PRB, and in addition to the decrease in the overall expression level of PR in endometriosis, the PRA/PRB ratio is also higher than that of normal endometrium. In fact, progesterone acts primarily on target genes through PRB, which antagonizes estrogen-induced endometrial epithelial cell proliferation through PRA [30]. This abnormal progesterone receptor change may affect the normal decidualization of the endometrium, making the endometrium less receptive, and may be one of the causes of infertility in patients with endometriosis [31]. In addition, the level of estrogen in endometriosis is abnormally high, and the enzymes related to estrogen synthesis, such as aromatase and HSD17β1 (Hydroxysteroid 17-Beta Dehydrogenase 1), are also highly expressed or increased in endometriosis lesions. In a normally functioning endometrium, ERα expression is more predominant than ERβ. However, studies on ovarian endometriosis have found a reduced ERα/ERβ ratio [32]. Abnormally high estrogen and progesterone resistance increases the proliferation of ectopic endometrial cells and exerts anti-apoptotic effects.

Peritoneal macrophages and neutrophils are overactive in the peritoneal cavity as a result of dysregulation of the immune response to endometriotic lesions. Ectopic lesions are characterized by elevated expression of genes associated with cytokine–cytokine receptor interactions, immune cell recruitment, and cellular adhesion [33,34]. The number of CD8+ T lymphocytes in ectopic endometrial tissue is higher than in eutopic endometrial tissue [35]. It has been shown that patients with endometriosis have higher TGF-β levels in peritoneal fluid compared with healthy women [36]. TGF-β is one of the inflammatory mediators released by mast cells, which can promote the expression of fibrotic factors and mediate epithelial–mesenchymal transition [37], promoting the transformation of mesothelial cells into fibroblasts [38]. TGF-β may be involved in the differentiation of T cells and stimulate the release of IL-17 and IL-10, leading to lesion formation [39,40]. Additionally, immune cells create a pro-angiogenic and pro-neurogenic peritoneal microenvironment in endometriosis [41]. For example, macrophages release nerve growth factor (NGF), which promotes the development of pain-related receptors, leading to clinical symptoms of pain in patients [42]. In the following, we will systematically explore the specific mechanisms by which RCD is involved in the dysregulation of the immune–inflammatory system in endometriosis.

## 3. Regulated Cell Death Mechanisms in Endometriosis

### 3.1. Apoptosis in Endometriosis

**Figure 1:**
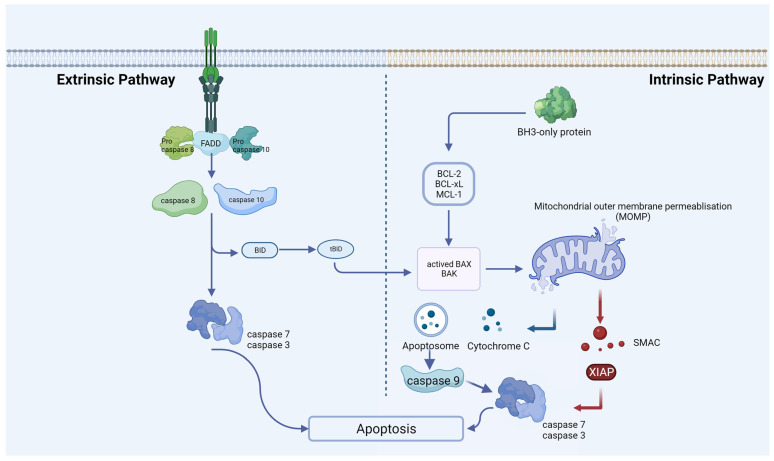
Extrinsic and intrinsic apoptotic pathways. An extrinsic pathway (death receptor-induced pathway) or an intrinsic pathway (mitochondrial or BCL-2-regulated pathway) can induce apoptosis. Caspases 8 and 10 undergo activation through the extrinsic pathway, a process initiated upon activation of death receptors situated at the plasma membrane. Various stress stimuli activate the intrinsic pathway, leading to mitochondrial outer-membrane permeabilization (MOMP), the release of cytochrome C, the formation of the apoptosome, and subsequent activation of caspase-9. At the proteolytic activation step of the effector caspases-3 and -7, both apoptotic pathways converge after the initiator caspases-8 and -9 are activated.

In 1972, Kerr and colleagues identified apoptosis, noting the morphological similarity of cell demise across various pathological conditions and normal tissue contexts [43]. This is the most well-characterized form of RCD, causing shrinkage, nuclear chromatin condensation, and fragmentation of the nucleus [44]. It is essential for disease prevention to maintain a balance between apoptosis and cell proliferation [45]. Cells with irreversible DNA damage are also removed by apoptosis as part of the immune system’s defense against infections [46]. By recognizing, uptaking, and degrading intact cells, apoptosis protects tissue from inflammation without releasing harmful contents. Rather than being a death mode, apoptosis is more like a mechanism for clearing out cells. Apoptosis is usually catalyzed by the proteolytic cleavage of thousands of proteins through the enzymatic activity of effector caspases like caspase 3 (Figure 1) [47]. Moreover, apoptosis can also be activated by granzyme contained within cytotoxic granules in T-cells or NK cells and through perforin-mediated pore formation in target cells [48]. In most mammalian cells, the increase in mitochondrial outer membrane permeability and the release of cytochrome c into the cytosol are key nodes that trigger apoptosis, which is regulated by pro-apoptotic and anti-apoptotic factors of the BCL-2 family [49]. Mitochondrial dysfunction can lead to impaired apoptosis. For example, in endometriotic tissues, CHCHD2 (Coiled-Coil-Helix-Coiled-Coil-Helix Domain Containing 2) expression may contribute to the pathogenesis of endometriosis through its regulation of mitochondria-mediated apoptosis [50]. Some scholars have confirmed that although the expression of apoptosis-related genes varies depending on the pathological type, there is overexpression of anti-apoptotic factors and insufficient expression of pro-apoptotic factors in endometriosis [51,52]. Another extrinsic cell death pathway is achieved through pro-apoptotic receptors such as Fas, TNF, TRAIL (TNF-related apoptosis-inducing ligand), etc. [53]. In endometriosis, apoptosis seems to be a protection mechanism. Estrogen and progesterone are also involved in this process. For endometriosis, estrogen mainly inhibits apoptosis through protein kinases, NF-kB, SRC-1, and other signaling pathways. Han and his colleagues discovered that ERβ can interact with cellular apoptotic machinery in the cytoplasm to inhibit TNF-a-induced apoptosis [54]. This mechanism may help endometriosis lesions evade endogenous immune surveillance. The mechanism by which progesterone regulates endometriosis apoptosis is relatively complex. In vivo experiments, normal women supplemented with progesterone at the late secretory phase can play an anti-endometrial apoptosis role, while in vitro experiments, progesterone can induce endometrial apoptosis [55]. The specific mechanism still needs more experimental verification. Contrary to endometrial cells, which undergo reduced apoptosis, ovarian granulosa cells of endometriosis patients undergo increased apoptosis. Consequently, folliculogenesis, oocyte and embryo quality, and IVF (in vitro fertilization) outcomes could be adversely affected [56,57]. Interestingly, it was found that when follicular fluid from patients with endometriosis-associated infertility was used for granulosa cell culture in patients with simple tubal infertility, the level of granulosa cell apoptosis was significantly increased, suggesting that there is a pro-granulosa cell apoptotic phenotype in follicular fluid from patients with endometriosis [58]. Additionally, elevated apoptosis was also found in cumulus cells from patients with ovarian endometrioma [59]. The exchange of material and signals between the cumulus cell and the oocytes is necessary for the maturation and ovulation of the oocytes. It is reasonable to speculate that infertility in patients with endometriosis may be associated with increased apoptosis of the cumulus cell.

Our recent research report affirms that GRIK1 antisense RNA (GRIK1-AS1) is capable of attenuating the proliferation of endometrial stromal cells. This effect is achieved through the inhibition of cell-cycle processes and the facilitation of apoptosis [60]. Another article revealed that an exosomal lncRNA, HOTAIR (HOX Transcript Antisense RNA), inhibits endometrial stromal cell apoptosis through sponging miR-761 [61]. Apoptotic activity linked with miRNA has been implicated in the pathological processes underlying endometriosis. Illustratively, a recent examination focusing on miRNAs governing adhesion and apoptosis revealed a noteworthy elevation in the expression levels of miR-93-5p and miR-7-5p in the cohorts afflicted with deep infiltrating endometriosis and endometrioma, as opposed to those presenting with lesions of superficial peritoneal endometriosis. Perhaps these results could help identify differences between pathological phenotypes of endometriosis [62].

Apoptosis is controlled by NF-κB transcription factors in a wide range of cell types, whether they block apoptosis or induce it. Endometrial cells promoted miR-138 to induce exosome-mediated inflammation and apoptosis in endometriosis through the VEGF/NF-κB signaling pathway [63]. Reactivating endometriosis apoptosis to inhibit the progression of the lesion is also a new direction for the treatment of endometriosis in the future. Ectopic lesions treated with oleuropein displayed higher levels of caspase-3 cleavage. Oleuropein also reactivated apoptosis in ectopic lesions by inhibiting ERβ and suppressing mouse endometriosis progression [64]. In addition, apoptosis is also regulated by some epigenetic modifications. For example, the depletion of histone deacetylase 2, HDAC2, can significantly promote the apoptosis of endometriosis cells [65]. Among the many studies of RCD mechanisms and endometriosis, apoptosis is undoubtedly one of the most studied and intensively researched mechanisms. Reducing or even reversing the anti-apoptotic properties of endometriosis lesions may become a completely new approach to treating endometriosis in the future.

### 3.2. Pyroptosis in Endometriosis

As a form of lytically programmed cell death, pyroptosis is initiated by inflammasomes that detect contamination or perturbation within the cytosol. Caspases-1 (canonical pathway) or caspase-11/4/5 (non-canonical pathway) are activated, which cleave gasdermin D (GSDMD) [66]. The morphological manifestation of pyroptosis is cell swelling and rupture of the plasma membrane, causing a release of pro-inflammatory cytokines and cellular contents into the extracellular space [66]. Unlike apoptosis, pyroptosis preserves mitochondrial integrity and prevents cytochrome C leakage.

Inflammasomes are protein complexes that contain three main parts: receptor proteins, adaptor proteins (ASCs), and downstream caspases. Receptor proteins are divided into the NOD-like receptor (NLR) family and the PYHIN family. Inflammasomes are assembled in response to pathogen-associated molecular patterns (PAMPs) and endogenous damage-associated molecular patterns (DAMPs) [67]. There are four main prototypes of inflammasome sensors found to date—NLR family pyrin domain containing 1 (NLRP1), NLRC4 (NLR Family CARD Domain Containing 4), absent in melanoma-2 (AIM2), and NLRP3 (NOD-like receptor thermal protein domain associated protein 3) [68]. In the canonical pathway, when these canonical inflammasome sensors are activated, the majority of these sensors interact with the ASCs, which activate caspase 1. Caspase1 cleaves GSDMD into two fragments, one at the C-terminus and the other at the N-terminus, which causes pores in the cell membrane via lysin phosphoinositide/cardiolipin-containing liposomes and triggers pyroptosis [69]. Moreover, caspase-1 also matures pro-IL-1β and pro-IL-18 into IL-1β and IL-18, which are released through the necrotic membrane pores formed by the GSDMD N-terminal fragment [70]. Non-canonical inflammasome pathways are uniquely mediated by caspase 11 (mice) and caspase 4/5 (humans). These caspases can directly bind with LPS and conduct the cleavage of GSDMD. By contrast, non-canonical inflammasomes that activate caspase 4/5/11 proteolysis only GSDMD cannot activate IL-1β and IL-18 directly [71]. However, secondary GSDMD pore-induced membrane damage and NLRP3 activation result in cytokine maturation in addition to GSDMD processing. This process also leads to an inflammatory response.

In recent years, transcription factors have been shown to regulate pyroptosis in endometriosis. TRIM24 is a member of the three-gene sequence protein (TRIM) family and belongs to the transactivator. The TRIM24 receptor targets are located in the nucleus and affect their expression and function by regulating chromosomal remodeling-related proteins [72]. An inhibitory effect of TRIM24 was observed on the NLRP3/CASP1β-mediated pyroptosis and cell migration of human endometrial stromal cells. The upregulation of TRIM24 facilitated the ubiquitination of NLRP3 [73]. Another transcription factor, FoxA2, is expressed specifically in the glands of the uterus and is a critical regulator of postnatal uterine gland differentiation in mice [74]. It is reported that upregulation of FoxA2 (Forkhead Box A2) downregulates ERβ by transcriptionally inhibiting IGF2BP1, thereby repressing pyroptosis in endometriosis [75].

**Figure 2:**
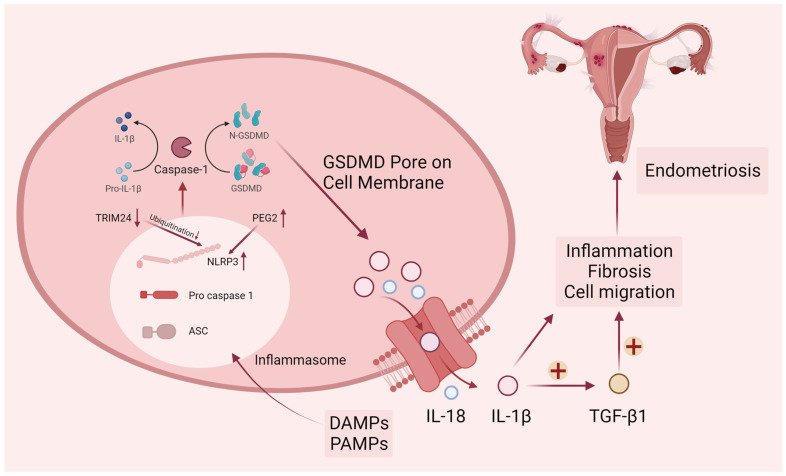
Canonical pyroptosis pathway in endometriosis. DAMPs (damage-associated molecular patterns) and PAMPs (pathogen-associated molecular patterns) elicit the activation of cytosolic canonical inflammasomes, notably NLRP3, leading to the subsequent cleavage of caspase-1. Following the activation of inflammatory caspases, the precursors pro-IL-1β, pro-IL-18, and GSDMD undergo cleavage. The N-terminal fragment of GSDMD (N-GSDMD) can form pores on the plasma membrane, facilitating the release of inflammatory mediators such as IL-1β and IL-18. The resultant pro-inflammatory microenvironment in endometriosis is conducive to the promotion of endometriotic cell migration and fibrosis. Upper arrows represent increased protein levels and lower arrows represent decreased protein levels.

Endometriosis can induce chronic pelvic inflammation and tissue fibrosis. During pathogenesis, PGE2-induced NLRP3/caspase1 pyroptosis plays a vital role in the invasion of endometriosis lesions. Huang et al. examined the expression level of pyroptosis-related proteins such as NLRP3, caspase-1, IL-1β, and IL-18 in endometriosis and found them significantly higher than normal endometrium [13]. In a bioinformatics study on endometriosis, researchers screened for pyroptosis genes that are closely related to endometriosis and used these genes to score pyroptosis levels in samples from public databases [76]. There is a strong correlation between higher levels of pyroptosis and more aggressive disease features, including epithelial–mesenchymal transition, angiogenesis, and impaired immunity [14]. Endometriosis relies heavily on new blood and vascular system formation to progress, so angiogenesis is essential in its progression [77,78,79]. NLRP3 inflammasome-mediated activation of pyroptosis can affect angiogenesis in endometriosis in a Notch1-dependent manner [80]. Fibrosis is the development of fibrous connective tissue in response to repeated tissue injury and repair, with myofibroblasts playing a key role in driving the fibrotic process. Once myofibroblasts are activated and produce a large amount of collagen extracellular matrix, they destroy the surrounding cellular structures. Fibrotic tissue often appears as scarring that is stiff and lacks blood vessels, complicating the surgical anatomy of endometriosis. Liu’s team demonstrated that aberrantly elevated lnc-MALAT1 (Metastasis Associated Lung Adenocarcinoma Transcript 1) in ectopic endometrium is associated with NLRP3-mediated pyroptosis and fibrosis, and lnc-MALAT1 sponges miR-141-3p to promote NLRP3 expression [81]. From the above results, it can be seen that pyroptosis regulates the pathological processes of endometriosis, such as inflammatory immune response, cell invasion, and fibrosis (Figure 2). In particular, the NLRP3-mediated pyroptosis pathway is involved in many mechanisms.

### 3.3. Ferroptosis in Endometriosis

Iron overload and lipid peroxidation are typical symptoms of ferroptosis, a type of iron-dependent cell death [82]. Several ferroptosis-inducing factors have been identified as influencing glutathione peroxidase, which eventually leads to decreased antioxidant capacity and lipid reactive oxygen species (ROS) accumulation in cells that ultimately causes oxidative cell death [83]. Morphologically, cells undergoing ferroptosis usually show necrosis-like morphological changes. These features include a loss of plasma membrane integrity, cytoplasmic swelling, swelling of cytoplasmic organelles, and moderate chromatin condensation [84]. Ferroptosis can also be accompanied by autophagosome development and detachment.

The system Xc-GSH-GPX4 pathway is a classic pathway for ferroptosis. System Xc- is a cystine/glutamate antiporter that exchanges extracellular cystine with intracellular glutamate [85]. Once cystine enters the cell, it is rapidly reduced, producing cysteine for glutathione biosynthesis. Glutathione plays a crucial role in intracellular antioxidant defense (GSH). An increase in oxidative stress and cell death can occur when GSH is depleted [86]. GPX4 is a member of the glutathione peroxidase (GPX) family. GPX4 is the only intracellular GPX used in the reduction of liposomal peroxides, which can convert lipid hydroperoxides into non-toxic lipid alcohols and prevent ferroptosis [87]. Intracellular GSH depletion and decreased activity of GPX4 occur during ferroptosis. Inhibitions of GPX4 activity prevent the reduction reaction mediated by GPX4 from metabolizing lipid peroxides, resulting in their accumulation [88].

**Figure 3:**
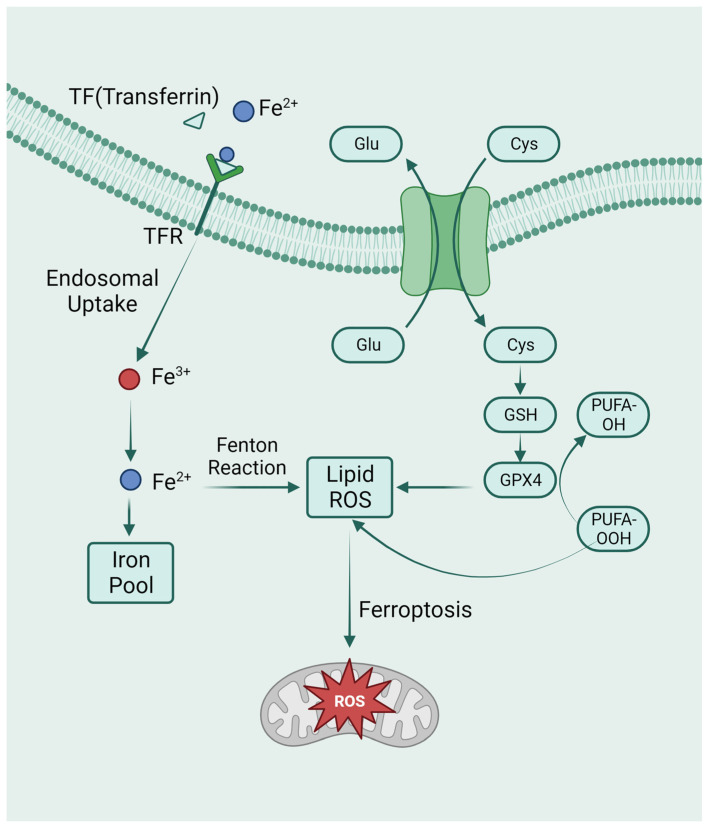
The metabolic pathways of ferroptosis. Intracellular glutamate (Glu) and extracellular cysteine (Cys) are transported via System Xc-, facilitating the incorporation of cysteine into glutathione (GSH) synthesis. Glutathione peroxidase 4 (GPX4) actively participates in the intracellular neutralization process, converting endogenous peroxidized polyunsaturated fatty acids (PUFAs-OOH) to their non-peroxidized counterparts (PUFAs-OH). This enzymatic activity culminates in the mitigation of reactive oxygen species (ROS) accumulation. Ferroptosis is caused by excess iron. Through TFR, circulating iron is combined with TF and enters cells. Iron in Fe^3+^ was deoxidized to iron in Fe^2+^. Ultimately, Fe^2+^ was released into a labile iron pool in the cytoplasm. Fenton reaction is a chain reaction between ferric ions (Fe^2+^) and hydrogen peroxide to catalyze the formation of OH radicals. The free Fe^2+^ in the iron pool participates in the Fenton reaction, generating ROS substances, and the accumulated ROS peroxidizes membrane lipids, resulting in loss of cell function and cell death.

Iron overload is another important feature of ferroptosis. The Fenton reaction, which results in non-enzymatic lipid peroxidation, regulates ferroptosis by producing lethal reactive oxygen species (ROS) [89]. As a cofactor for iron-containing enzymes, iron may also be essential for enzymatic lipid metabolism. Therefore, iron appears to play a vital role in ferroptosis, whether enzymatically or non-enzymatically, in the production of ROS (Figure 3). In addition to the above-mentioned GPX4-mediated classical ferroptosis pathway, the mitochondrial transmembrane channel VDAC (voltage-dependent anion channel) and the tumor suppressor gene P53 can also mediate ferroptosis [90,91].

In endometriotic lesions, erythrocyte degradation leads to iron accumulation [92,93]. Iron overload influences the preimplantation process of the endometriosis mouse embryo. Mechanically, iron overload can disrupt mitochondrial function by interfering with ATP production. Additionally, iron overload can induce intracellular ROS. Embryos cultured at higher iron concentrations showed lower rates of cleavage and blastocyst formation [94]. Treatment of mouse granulosa cells with follicular fluid from patients with endometriosis-associated infertility can induce ferroptosis, which hinders oocyte maturation by releasing exosomes [95]. These studies provide a new perspective on ferroptosis’s involvement in endometriosis-induced infertility. Some lncRNA can also regulate ferroptosis in endometriosis. For example, up-regulated ADAMTS9-AS1 (ADAM Metallopeptidase with Thrombospondin Type 1 Motif Antisense RNA 1) accelerates endometrial proliferation and migration by modulating miR-6516-5p/GPX4-dependent ferroptosis. ADAMTS9-AS1 increased ROS levels, and inhibition of this lncRNA significantly reduced GPX4 expression [96]. Ferroptosis is associated with endometriosis-derived clear cell carcinoma of the ovary (CCOC). Compared with the normal secretory endometrium, the expression of cysteine and glutathione synthesis pathway genes and the downregulation of iron antiporter were observed in CCOC [97]. According to another study, CD 10 negative endometriosis-derived mesenchymal stem cells expressed a high level of iron export proteins and were capable of transmitting iron to associated CCOC cells [98]. Significantly, the stroma may support the growth and development of tumor cells through iron transport and donation. Further characterization of the stromal phenotype may be a new direction in the study of malignant transformation in endometriosis.

As we have already mentioned above, one of the characteristics of endometriosis foci is myofibroblast-induced fibrosis and angiogenesis. Ferroptosis is also involved in these processes, and studies by Zhang et al. showed that ferroptosis inhibitors could reduce the proportion of myofibroblasts in endometriosis lesions and alleviate fibrosis [15]. Endometrial stromal cell ferroptosis in the ovarian endometrioma may promote angiogenesis [17]. Erastin, a ferroptosis inducer, can shrink endometriosis lesions, but the mechanism remains to be explored [99]. The MALAT1/miR-145-5p/MUC1 axis was involved in shrinking endometriotic lesions caused by erastin-induced ferroptosis [100]. Perhaps there are other regulatory mechanisms for the ameliorating effect of erastin on endometriosis, and this ferroptosis inhibitor can be applied to the drug treatment of endometriosis in the future.

### 3.4. Cuproptosis in Endometriosis

**Figure 4:**
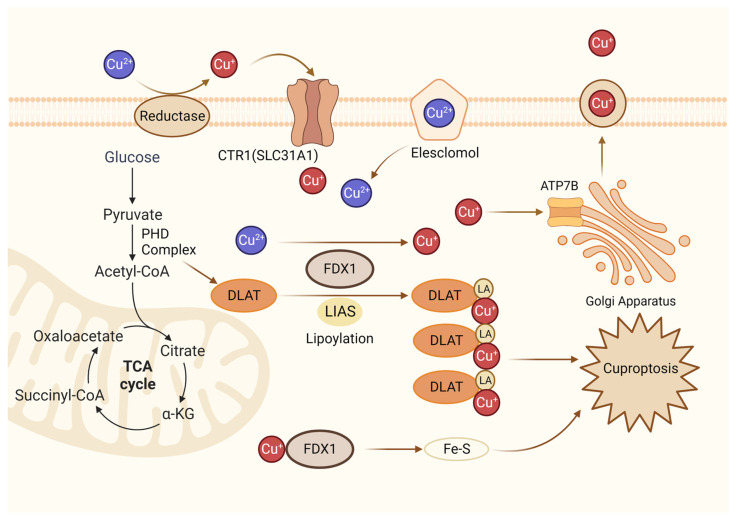
Diagrammatic representation of cuproptosis mechanism. Cuproptosis suppresses mitochondrial oxidative respiration through the inhibition of DLAT, a crucial constituent within the pyruvate dehydrogenase complex (PDH complex). Elesclomol binds extracellular Cu and transports it to intracellular compartments. Upon entry into the cell, Cu engages with lipoylated mitochondrial enzymes in the tricarboxylic acid cycle (TCA), such as DLAT. LIAS/FDX1 regulates protein lipoylation, facilitating mitochondrial protein aggregation and Fe-S cluster loss. As a result of these aberrant processes, proteotoxic stress occurs, and cells die. Lipoylation is a post-translational modification specific to mitochondrial proteins. LA: lipoic acid. DLAT: dihydrolipoyl transacetylase.

New research has revealed that copper-dependent cuproptosis is a non-apoptotic mode of cell death that regulates mitochondrial respiration. During cuproptosis, copper ions are combined with fatty acylated components in the tricarboxylic acid cycle (Figure 4). Consequently, fatty acylated proteins aggregate and iron-sulfur cluster proteins are reduced, resulting in protein toxicity stress and cell death [101]. FDX1(Ferredoxin 1) is a ferrite-reducing protein, which is the core molecule of cuproptosis. On the one hand, FDX1 can reduce Cu^2+^ to Cu^+^, which is more toxic, to induce cuproptosis. On the other hand, it can catalyze the lipacylation of pyruvate dehydrogenase core structural proteins [102]. According to a recent study, FDX1 mediates cuproptosis in endometriosis through the G6PD pathway, which inhibits the proliferation and metastasis of endometriosis cells [102]. It is still unclear how cuproptosis occurs in endometriosis, and research in this field holds great promise.

## 4. The Interplay between RCD Mechanisms in Endometriosis

Various forms of RCD exist, with intricate interactions and mutual influences observed among the complex pathways associated with each type of cell demise. In endometriosis and adenomyosis, iron overload inhibits cell proliferation and promotes autophagic cell death via PARP1 (Poly (ADP-Ribose) Polymerase 1)/SIRT1 (Sirtuin 1) signaling in endometriosis and adenomyosis [103]. Moreover, the autophagic cell death inducer rapamycin can increase iron content, reactive oxygen species, lipid peroxide production, and ferroptosis mitochondrial morphology, indicating autophagic cell death-dependent ferroptosis is involved in the development of endometriosis [104]. Although crosstalk between RCDs has not been widely studied in endometriosis, it is not difficult to see from the relevant studies of other diseases that it is a direction worth exploring. For example, in a study on pyroptosis and apoptosis, researchers found that channel-forming glycoprotein pannexin-1, but not GSDMD or GSDME, promotes NLRP3 inflammasome activation during caspase-8 or caspase-9-dependent apoptosis [105].

At present, a form of cell death called PAN-optosis has entered the field of vision of scientists. The concept of PAN-optosis was established based on the study of the interaction between inflammasome/pyroptosis, apoptosis, and necroptosis. As can be seen from its name, “P” stands for pyroptosis, “A” stands for apoptosis, and “N” stands for necroptosis [106]. This process is mainly implemented through a structure called “PAN-optosome.” PANoptosome is a multi-protein complex that provides a molecular scaffold that contains key proteins that activate pyroptosis, apoptosis, and programmed necrosis [107]. PAN-optosis is a unique, innate immune–inflammatory RCD pathway that is regulated by PANoptosome complexes upon sensing pathogens, PAMPs, DAMPs, or the cytokines produced downstream [108]. Proper PAN-optosis can trigger an infiltration of immune cells, which can clear infectious agents. However, if regulation goes wrong, excessive PAN-optosis can also lead to harmful inflammation and tissue damage. Therefore, the balance regulation of PAN-optosis is the subject of future research for endometriosis treatment.

## 5. RCD Therapeutic Perspectives in Endometriosis

In recent years, new advances have been made in immunotherapy for endometriosis. According to a recent study, extracellular adenosine triphosphate (eATP), an important inflammatory mediator, alleviates the dysfunction of macrophages caused by endometriosis and promotes the recruitment of macrophages [109]. In addition, some natural ingredients from plants and animals have also been extracted by scientists for research related to the treatment of endometriosis. Quercetin has antiproliferative and anti-inflammatory effects on endometriosis mice. The mRNA expression of CCND1 (Cyclin D1) was significantly reduced after intraperitoneal injection of quercetin in the diseased mice. After knocking out the CCND1 mRNA, the proliferation of endometriosis cell lines is attenuated, the sub-G0/G1 cell cycle is stopped, and apoptosis is increased [110]. Bufalin is an endogenous cardiotonic steroid found in toad venom as well as in healthy human plasma, with anti-tumor properties in several types of cancer [111]. It was reported that bufalin-induced disruption of the SRC-1 (steroid receptor coactivator 1) isoform/ERβ axis might induce apoptosis, pyroptosis, and endoplasmic reticulum stress signaling in endometriotic lesions, suppressing endometriosis. Mechanically, bufalin disrupted the functional axis of SRC-1 isoform/ERβ by increasing SRC-1 isoform protein stability, hyperactivating the transcriptional activity of the SRC-1 isoform, and degrading the ERβ protein by proteasome 26S subunit, non-ATPase 2 in endometriotic lesions [112]. Ferroptosis promotes endometriosis progression by impairing macrophage phagocytosis and producing more pro-angiogenic factors. Baicalein is a potential anti-ferroptosis compound that increases GPX4 expression, significantly inhibits ferroptosis, and restores phagocytosis in THP-1 cells (a human leukemia monocytic cell line) in vitro [113]. Açai Berry administration was able to modulate autophagy, oxidative stress, and apoptosis in mouse endometriosis models [114]. Alpinumisoflavone is an isoflavonoid extracted from fruit that inhibits cell migration and proliferation and leads to cell apoptosis in endometriosis cell lines [115].

Dienogest is currently the first-line oral drug for the treatment of endometriosis. In women with ovarian endometrioma, dienogest can reduce the size of ovarian cysts [116]. It is effective in reducing endometriosis-related symptoms after 6 and 12 months of treatment [117]. Dienogest is well tolerated and has a better response to endoscopy-related pain than drugs such as danazol and leuprolide. In studies on RCD with dienogest, scientists found this 19-nortestosterone derivative can reduce NLRP3 inflammasome-mediated IL-1β production through autophagy induction [118]. Furthermore, dienogest treatment of endometriotic cells suppresses AKT and ERK1/2 activity, thereby, in turn, inhibiting mTOR, inducing autophagy, and promoting apoptosis [119]. Research about melatonin discovered that the combination of melatonin and dienogest effectively inhibited the proliferation of endometriotic cells due to melatonin-induced apoptosis [120]. Although the combination of melatonin and dienogest in the treatment of endometriosis still needs large-scale clinical studies to confirm its effectiveness, there is no doubt that this provides a new idea for the treatment of endometriosis.

Traditional Chinese medicine (TCM) has a rich theoretical basis and clinical application for endometriosis treatment. Bushen Wenyang Huayu Decoction, a compound Chinese medicine preparation, inhibits autophagy by up-regulating SIRT1 (Sirtuin 1) and down-regulating FoXO-1 (Forkhead box protein O) expression in endometriosis via the SIRT1-FoXO-1 signaling pathway [121]. In recent years, the therapeutic role of targeted nanomaterials in endometriosis has been further explored. A novel form of polymer-based NP gene delivery platform consisting of polyethyleneimine (PEI) conjugated to stearic acid (SA) and nucleotides (DNA/siRNAs) and enclosed by hyaluronic acid (HA) was invented to alleviate endometriosis by inducing cell death [122]. Perhaps in future studies, nanomaterials that specifically induce RCD can be designed to inhibit the growth of endometriosis.

The preceding discourse has detailed the discussion concerning naturally occurring constituents that exhibit the capacity to modulate the advancement of endometriotic lesions via RCD. The exploration of natural plant-derived constituents presents a novel avenue for dietary intervention for endometriosis. Notably, polyphenolic compounds emerge as prospective candidates for dietary therapy in endometriosis, owing to their demonstrated ability to induce apoptotic processes in both endometriotic cell lines and murine models of endometriosis [123]. Another study revealed that high-fat diet-induced apoptosis may be associated with endometriosis progression. Specifically, the number of lesions in the high-fat diet-fed endometriosis model mice was significantly higher than that in the normal diet-fed model mice. In the high-fat diet endometriosis model mice, the level of apoptosis was significantly reduced, which may be one of the reasons for the increased formation of lesions [124]. However, the effect of diet-regulated RCD on endometriosis is still in its infancy. Indeed, endometriosis is a complex disease, and there is a lack of large-scale prospective studies confirming the palliative effect of certain diet therapies on endometriosis pathologic progression and clinical symptoms. Diet-modulated RCD may be a new research direction for the treatment of endometriosis in the future.

## 6. Conclusions and Future Directions

Endometriosis is a disease with significant heterogeneity. There may be a discrepancy between the severity of the lesion and the patient’s perceived symptoms. Since endometriosis seriously affects women’s reproductive function and brings a heavy burden to women’s psychology and physiology, it is important to explore the pathogenesis and treatment of this disease. In this article, we summarize the basic mechanisms of apoptosis, pyroptosis, ferroptosis, and autophagy, as well as the main signaling molecules and pathways involved in the pathogenesis of endometriosis. However, the mechanism of some RCDs, such as necroptosis, anoikis, and cuproptosis in endometriosis, is still poorly studied. Although some researchers have preliminarily explored the phenotypes of necroptosis and cuproptosis in endometriosis through bioinformatics methods, the upstream and downstream molecules and the effects of this cell death mode on the immune infiltration environment of endometriosis still need to be confirmed by specific molecular biology and animal experiments.

In this article, we have mentioned that RCD-related immunotherapy can be used for endometriosis treatment. At present, most RCD-related immunotherapies are mainly directed at tumors, and there are few studies on immunotherapy for non-tumor diseases. We outlined the critical role of mitochondria in apoptosis above. In endometriosis, the reduced function of NK cells to kill ectopic endometrial cells can lead to ectopic endometrial adhesion and proliferation, which in turn leads to the immune escape of ectopic endometrial cells [64]. BH3 mimetics are a class of novel anti-tumor drugs targeting Bcl-2 family proteins that can mimic the BH3 domain of BH3-only proteins, interact with Bcl-2 family protein members, replace and release pro-apoptotic proteins, and induce apoptosis, thereby achieving anti-tumor effects [65,66]. The synergistic effect of BH3 mimetic and NK cells enhances mitochondrial apoptosis in tumor cells [67]. In the future, the mechanism of BH3 mimetics to enhance NK cell killing in endometriosis may be further explored to provide a reference for immunotherapy for endometriosis.

Endometriosis is a benign disease, but it has some invasive and aggressive features and an immune microenvironment similar to malignancy [125]. For example, in a study of the treatment of triple-negative breast cancer, researchers found that tumor tissues had a different metabolic phenotype of ferroptosis from normal tissues through metabolomics, and inhibition of the key ferroptosis protein GPX4 could effectively enhance cellular anti-tumor immunity [68]. At present, metabolomics research on endometriosis is still in its infancy, and many RCDs are involved in the metabolic regulation of the body, which makes us wonder if there is also a unique metabolic phenotype associated with a certain RCD in endometriosis. Can endometriosis be reversed by inhibiting or promoting the synthesis of RCD-related metabolites? Endometriosis still has a broad research prospect in the field of RCD, and it is believed that clarifying the complex molecular regulatory mechanism of RCD can provide a better method for the treatment of endometriosis.

## References

[1] Taylor H.S., Kotlyar A.M., Flores V.A. (2021). Endometriosis is a chronic systemic disease: Clinical challenges and novel innovations. Lancet.

[2] Peiris A.N., Chaljub E., Medlock D. (2018). Endometriosis. JAMA.

[3] de Ziegler D., Borghese B., Chapron C. (2010). Endometriosis and infertility: Pathophysiology and management. Lancet.

[4] McCallion A., Nasirzadeh Y., Lingegowda H., Miller J.E., Khalaj K., Ahn S., Monsanto S.P., Bidarimath M., Sisnett D.J., Craig A.W. (2022). Estrogen mediates *the* inflammatory role of mast cells in endometriosis pathophysiology. Front. Immunol..

[5] Carbone G., Nelson K., Baumgartner C., Bode A.M., Takahashi A., Chefetz I. (2023). Endometriosis: Cell Death and Cell Signaling Machinery. Endocrinology.

[6] Warner T.F. (1972). Apoptosis. Lancet.

[7] Sheng S.Y., Li J.M., Hu X.Y., Wang Y. (2023). Regulated cell death pathways in cardiomyopathy. Acta Pharmacol. Sin..

[8] Sanz A.B., Sanchez-Niño M.D., Ramos A.M., Ortiz A. (2023). Regulated cell death pathways in kidney disease. Nat. Rev. Nephrol..

[9] Del Re D.P., Amgalan D., Linkermann A., Liu Q., Kitsis R.N. (2019). Fundamental Mechanisms of Regulated Cell Death and Implications for Heart Disease. Physiol. Rev..

[10] Bedoui S., Herold M.J., Strasser A. (2020). Emerging connectivity of programmed cell death pathways and its physiological implications. Nat. Rev. Mol. Cell Biol..

[11] Gao W., Wang X., Zhou Y., Wang X., Yu Y. (2022). Autophagy, ferroptosis, pyroptosis, and necroptosis in tumor immunotherapy. Signal Transduct. Target. Ther..

[12] Tang D., Kang R., Berghe T.V., Vandenabeele P., Kroemer G. (2019). The molecular machinery of regulated cell death. Cell Res..

[13] Huang Y., Li R., Hu R., Yao J., Yang Y. (2022). PEG2-Induced Pyroptosis Regulates the Expression of HMGB1 and Promotes hEM15A Migration in Endometriosis. Int. J. Mol. Sci..

[14] Guo Q., Zhou C., Xiang Y., Liang X. (2023). Pyroptosis orchestrates immune responses in endometriosis. Int. Immunopharmacol..

[15] Zhang Y., Liu X., Deng M., Xu C., Zhang Y., Wu D., Tang F., Yang R., Miao J. (2022). Ferroptosis induced by iron overload promotes fibrosis in ovarian endometriosis and is related to subpopulations of endometrial stromal cells. Front. Pharmacol..

[16] Wang X., Wei Y., Wei F., Kuang H. (2023). Regulatory mechanism and research progress of ferroptosis in obstetrical and gynecological diseases. Front. Cell Dev. Biol..

[17] Li G., Lin Y., Zhang Y., Gu N., Yang B., Shan S., Liu N., Ouyang J., Yang Y., Sun F. (2022). Endometrial stromal cell ferroptosis promotes angiogenesis in endometriosis. Cell Death Discov..

[18] Peinado F.M., Olivas-Martínez A., Iribarne-Durán L.M., Ubiña A., León J., Vela-Soria F., Fernández-Parra J., Fernández M.F., Olea N., Freire C. (2023). Cell cycle, apoptosis, cell differentiation, and lipid metabolism gene expression in endometriotic tissue and exposure to parabens and benzophenones. Sci. Total Environ..

[19] Miura R., Yokoi A., Matsumoto T., Oguri Y., Hashimura M., Tochimoto M., Kajita S., Saegusa M. (2019). Nodal induces apoptosis and inhibits proliferation in ovarian endometriosis-clear cell carcinoma lesions. BMC Cancer.

[20] Harada T., Kaponis A., Iwabe T., Taniguchi F., Makrydimas G., Sofikitis N., Paschopoulos M., Paraskevaidis E., Terakawa N. (2004). Apoptosis in human endometrium and endometriosis. Hum. Reprod. Update.

[21] Sampson J.A. (1927). Metastatic or Embolic Endometriosis, due to the Menstrual Dissemination of Endometrial Tissue into the Venous Circulation. Am. J. Pathol..

[22] Jeljeli M., Riccio L.G.C., Chouzenoux S., Moresi F., Toullec L., Doridot L., Nicco C., Bourdon M., Marcellin L., Santulli P. (2020). Macrophage Immune Memory Controls Endometriosis in Mice and Humans. Cell Rep..

[23] Gajbhiye R.K. (2023). Endometriosis and inflammatory immune responses: Indian experience. Am. J. Reprod. Immunol..

[24] Sugihara K., Kobayashi Y., Suzuki A., Tamura N., Motamedchaboki K., Huang C.T., Akama T.O., Pecotte J., Frost P., Bauer C. (2014). Development of pro-apoptotic peptides as potential therapy for peritoneal endometriosis. Nat. Commun..

[25] Klemmt P.A., Carver J.G., Kennedy S.H., Koninckx P.R., Mardon H.J. (2006). Stromal cells from endometriotic lesions and endometrium from women with endometriosis have reduced decidualization capacity. Fertil. Steril..

[26] Delbandi A.A., Mahmoudi M., Shervin A., Akbari E., Jeddi-Tehrani M., Sankian M., Kazemnejad S., Zarnani A.H. (2013). Eutopic and ectopic stromal cells from patients with endometriosis exhibit differential invasive, adhesive, and proliferative behavior. Fertil. Steril..

[27] Bergqvist I.A. (1995). Hormonal regulation of endometriosis and the rationales and effects of gonadotrophin-releasing hormone agonist treatment: A review. Hum. Reprod..

[28] Asghari S., Valizadeh A., Aghebati-Maleki L., Nouri M., Yousefi M. (2018). Endometriosis: Perspective, lights, and shadows of etiology. Biomed. Pharmacother..

[29] Leeners B., Farquhar C.M. (2019). Benefits of pregnancy on endometriosis: Can we dispel the myths?. Fertil. Steril..

[30] Marquardt R.M., Kim T.H., Shin J.H., Jeong J.W. (2019). Progesterone and Estrogen Signaling in the Endometrium: What Goes Wrong in Endometriosis?. Int. J. Mol. Sci..

[31] Li X., Jin J., Long X., Weng R., Xiong W., Liang J., Liu J., Sun J., Cai X., Zhang L. (2023). METTL3-regulated m6A modification impairs the decidualization of endometrial stromal cells by regulating YTHDF2-mediated degradation of FOXO1 mRNA in endometriosis-related infertility. Reprod. Biol. Endocrinol..

[32] Huhtinen K., Ståhle M., Perheentupa A., Poutanen M. (2012). Estrogen biosynthesis and signaling in endometriosis. Mol. Cell Endocrinol..

[33] Ma J., Zhang L., Zhan H., Mo Y., Ren Z., Shao A., Lin J. (2021). Single-cell transcriptomic analysis of endometriosis provides insights into fibroblast fates and immune cell heterogeneity. Cell Biosci..

[34] Dai Y., Lin X., Liu N., Shi L., Zhuo F., Huang Q., Gu W., Zhao F., Zhang Y., Zhang Y. (2023). Integrative analysis of transcriptomic and metabolomic profiles reveals abnormal phosphatidylinositol metabolism in follicles from endometriosis-associated infertility patients. J. Pathol..

[35] Kisovar A., Becker C.M., Granne I., Southcombe J.H. (2023). The role of CD8+ T cells in endometriosis: A systematic review. Front. Immunol..

[36] Young V.J., Ahmad S.F., Duncan W.C., Horne A.W. (2017). The role of TGF-β in the pathophysiology of peritoneal endometriosis. Hum. Reprod. Update.

[37] Mwaura A.N., Riaz M.A., Maoga J.B., Mecha E., Omwandho C.O.A., Scheiner-Bobis G., Meinhold-Heerlein I., Konrad L. (2022). Activin A Modulates Betaglycan Shedding via the ALK4-SMAD3-Dependent Pathway in Endometriotic Cells. Biomolecules.

[38] Arablou T., Aryaeian N., Khodaverdi S., Kolahdouz-Mohammadi R., Moradi Z., Rashidi N., Delbandi A.A. (2021). The effects of resveratrol on the expression of VEGF, TGF-β, and MMP-9 in endometrial stromal cells of women with endometriosis. Sci. Rep..

[39] Lurje I., Gaisa N.T., Weiskirchen R., Tacke F. (2023). Mechanisms of organ fibrosis: Emerging concepts and implications for novel treatment strategies. Mol. Asp. Med..

[40] Ghoreschi K., Laurence A., Yang X.P., Tato C.M., McGeachy M.J., Konkel J.E., Ramos H.L., Wei L., Davidson T.S., Bouladoux N. (2010). Generation of pathogenic T(H)17 cells in the absence of TGF-β signalling. Nature.

[41] McKinnon B.D., Evers J., Bersinger N.A., Mueller M.D. (2013). Induction of the neurokinin 1 receptor by TNFα in endometriotic tissue provides the potential for neurogenic control over endometriotic lesion growth. J. Clin. Endocrinol. Metab..

[42] Velho R.V., Taube E., Sehouli J., Mechsner S. (2021). Neurogenic Inflammation in the Context of Endometriosis-What Do We Know?. Int. J. Mol. Sci..

[43] Kerr J.F., Wyllie A.H., Currie A.R. (1972). Apoptosis: A basic biological phenomenon with wide-ranging implications in tissue kinetics. Br. J. Cancer.

[44] Saraste A., Pulkki K. (2000). Morphologic and biochemical hallmarks of apoptosis. Cardiovasc. Res..

[45] Hockenbery D. (1995). Defining apoptosis. Am. J. Pathol..

[46] Savill J. (1994). Apoptosis in disease. Eur. J. Clin. Investig..

[47] Cohen G.M. (1997). Caspases: The executioners of apoptosis. Biochem. J..

[48] Carneiro B.A., El-Deiry W.S. (2020). Targeting apoptosis in cancer therapy. Nat. Rev. Clin. Oncol..

[49] Delbridge A.R., Grabow S., Strasser A., Vaux D.L. (2016). Thirty years of BCL-2: Translating cell death discoveries into novel cancer therapies. Nat. Rev. Cancer.

[50] Ren Y.Q., Wang X.R., Guo J.Y., Wang D., Li X.H., Cheng X.M., Wang X.G. (2022). CHCHD2 Regulates Mitochondrial Function and Apoptosis of Ectopic Endometrial Stromal Cells in the Pathogenesis of Endometriosis. Reprod. Sci..

[51] Gebel H.M., Braun D.P., Tambur A., Frame D., Rana N., Dmowski W.P. (1998). Spontaneous apoptosis of endometrial tissue is impaired in women with endometriosis. Fertil. Steril..

[52] Dmowski W.P., Gebel H., Braun D.P. (1998). Decreased apoptosis and sensitivity to macrophage mediated cytolysis of endometrial cells in endometriosis. Hum. Reprod. Update.

[53] Cullen S.P., Martin S.J. (2015). Fas and TRAIL ‘death receptors’ as initiators of inflammation: Implications for cancer. Semin. Cell Dev. Biol..

[54] Han S.J., Jung S.Y., Wu S.P., Hawkins S.M., Park M.J., Kyo S., Qin J., Lydon J.P., Tsai S.Y., Tsai M.J. (2015). Estrogen Receptor β Modulates Apoptosis Complexes and the Inflammasome to Drive the Pathogenesis of Endometriosis. Cell.

[55] Béliard A., Noël A., Foidart J.M. (2004). Reduction of apoptosis and proliferation in endometriosis. Fertil. Steril..

[56] Fujino K., Yamashita Y., Hayashi A., Asano M., Morishima S., Ohmichi M. (2008). Survivin gene expression in granulosa cells from infertile patients undergoing in vitro fertilization-embryo transfer. Fertil. Steril..

[57] Corachán A., Pellicer N., Pellicer A., Ferrero H. (2021). Novel therapeutic targets to improve IVF outcomes in endometriosis patients: A review and future prospects. Hum. Reprod. Update.

[58] Chen L., Ni Z.X., Cai Z.L., Cheng W., Sun S., Yu C.Q., JinYu (2021). The Mechanism Exploration of Follicular Fluids on Granulose Cell Apoptosis in Endometriosis-Associated Infertility. Biomed. Res. Int..

[59] Sreerangaraja Urs D.B., Wu W.H., Komrskova K., Postlerova P., Lin Y.F., Tzeng C.R., Kao S.H. (2020). Mitochondrial Function in Modulating Human Granulosa Cell Steroidogenesis and Female Fertility. Int. J. Mol. Sci..

[60] Liu W., Hu B., Wang X., Huang E., Chen X., Chen L. (2023). GRIK1-AS1 deficiency accelerates endometriosis progression by boosting DNMT1-dependent SFRP1 promoter methylation in endometrial stromal cells. J. Gene Med..

[61] Zhang L., Yu Z., Qu Q., Li X., Lu X., Zhang H. (2022). Exosomal lncRNA HOTAIR Promotes the Progression and Angiogenesis of Endometriosis via the miR-761/HDAC1 Axis and Activation of STAT3-Mediated Inflammation. Int. J. Nanomed..

[62] Antonio L.G.L., Meola J., Rosa E.S.A., Nogueira A.A., Candido Dos Reis F.J., Poli-Neto O.B., Rosa E.S.J.C. (2023). Altered Differential Expression of Genes and microRNAs Related to Adhesion and Apoptosis Pathways in Patients with Different Phenotypes of Endometriosis. Int. J. Mol. Sci..

[63] Zhang A., Wang G., Jia L., Su T., Zhang L. (2019). Exosome-mediated microRNA-138 and vascular endothelial growth factor in endometriosis through inflammation and apoptosis via the nuclear factor-κB signaling pathway. Int. J. Mol. Med..

[64] Park Y., Cho Y.J., Sung N., Park M.J., Guan X., Gibbons W.E., O’Malley B.W., Han S.J. (2022). Oleuropein suppresses endometriosis progression and improves the fertility of mice with endometriosis. J. Biomed. Sci..

[65] Mai H., Liao Y., Luo S.F., Wei K.Y., Yang F., Shi H.J. (2021). Histone deacetylase HDAC2 silencing prevents endometriosis by activating the HNF4A/ARID1A axis. J. Cell. Mol. Med..

[66] Rao Z., Zhu Y., Yang P., Chen Z., Xia Y., Qiao C., Liu W., Deng H., Li J., Ning P. (2022). Pyroptosis in inflammatory diseases and cancer. Theranostics.

[67] Li Y., Yuan Y., Huang Z.X., Chen H., Lan R., Wang Z., Lai K., Chen H., Chen Z., Zou Z. (2021). GSDME-mediated pyroptosis promotes inflammation and fibrosis in obstructive nephropathy. Cell Death Differ..

[68] Vande Walle L., Lamkanfi M. (2016). Pyroptosis. Curr. Biol..

[69] Burdette B.E., Esparza A.N., Zhu H., Wang S. (2021). Gasdermin D in pyroptosis. Acta Pharm. Sin. B.

[70] Chen J., Chen Z.J. (2018). PtdIns4P on dispersed trans-Golgi network mediates NLRP3 inflammasome activation. Nature.

[71] He W.T., Wan H., Hu L., Chen P., Wang X., Huang Z., Yang Z.H., Zhong C.Q., Han J. (2015). Gasdermin D is an executor of pyroptosis and required for interleukin-1β secretion. Cell Res..

[72] Yu T., Gan S., Zhu Q., Dai D., Li N., Wang H., Chen X., Hou D., Wang Y., Pan Q. (2019). Modulation of M2 macrophage polarization by the crosstalk between Stat6 and Trim24. Nat. Commun..

[73] Hang Y., Tan L., Chen Q., Liu Q., Jin Y. (2021). E3 ubiquitin ligase TRIM24 deficiency promotes NLRP3/caspase-1/IL-1β-mediated pyroptosis in endometriosis. Cell Biol. Int..

[74] Kelleher A.M., Peng W., Pru J.K., Pru C.A., DeMayo F.J., Spencer T.E. (2017). Forkhead box a2 (FOXA2) is essential for uterine function and fertility. Proc. Natl. Acad. Sci. USA.

[75] Feng Y., Tan B., Dong H., Zheng L. (2023). FoxA2 represses ERβ-mediated pyroptosis in endometriosis by transcriptionally inhibiting IGF2BP1. Exp. Cell Res..

[76] Sun J., Gan L., Sun J. (2022). Identification and Validation of Three m6A Regulators: FTO, HNRNPC, and HNRNPA2B1 as Potential Biomarkers for Endometriosis. Genes.

[77] Samimi M., Pourhanifeh M.H., Mehdizadehkashi A., Eftekhar T., Asemi Z. (2019). The role of inflammation, oxidative stress, angiogenesis, and apoptosis in the pathophysiology of endometriosis: Basic science and new insights based on gene expression. J. Cell Physiol..

[78] Nanda A., K T., Banerjee P., Dutta M., Wangdi T., Sharma P., Chaudhury K., Jana S.K. (2020). Cytokines, Angiogenesis, and Extracellular Matrix Degradation are Augmented by Oxidative Stress in Endometriosis. Ann. Lab. Med..

[79] Laschke M.W., Menger M.D. (2007). In vitro and in vivo approaches to study angiogenesis in the pathophysiology and therapy of endometriosis. Hum. Reprod. Update.

[80] Zhang M., Shi Z., Peng X., Cai D., Peng R., Lin Y., Dai L., Li J., Chen Y., Xiao J. (2023). NLRP3 inflammasome-mediated Pyroptosis induce Notch signal activation in endometriosis angiogenesis. Mol. Cell Endocrinol..

[81] Xu Y., Liu H., Xiong W., Peng Y., Li X., Long X., Jin J., Liang J., Weng R., Liu J. (2023). A novel mechanism regulating pyroptosis-induced fibrosis in endometriosis via lnc-MALAT1/miR-141-3p/NLRP3 pathway†. Biol. Reprod..

[82] Yang F., Xiao Y., Ding J.H., Jin X., Ma D., Li D.Q., Shi J.X., Huang W., Wang Y.P., Jiang Y.Z. (2023). Ferroptosis heterogeneity in triple-negative breast cancer reveals an innovative immunotherapy combination strategy. Cell Metab..

[83] Tang D., Chen X., Kang R., Kroemer G. (2021). Ferroptosis: Molecular mechanisms and health implications. Cell Res..

[84] Liang D., Minikes A.M., Jiang X. (2022). Ferroptosis at the intersection of lipid metabolism and cellular signaling. Mol. Cell.

[85] Li F.J., Long H.Z., Zhou Z.W., Luo H.Y., Xu S.G., Gao L.C. (2022). System X(c)(-)/GSH/GPX4 axis: An important antioxidant system for the ferroptosis in drug-resistant solid tumor therapy. Front. Pharmacol..

[86] Wang L., Liu Y., Du T., Yang H., Lei L., Guo M., Ding H.F., Zhang J., Wang H., Chen X. (2020). ATF3 promotes erastin-induced ferroptosis by suppressing system Xc(). Cell Death Differ..

[87] Bersuker K., Hendricks J.M., Li Z., Magtanong L., Ford B., Tang P.H., Roberts M.A., Tong B., Maimone T.J., Zoncu R. (2019). The CoQ oxidoreductase FSP1 acts parallel to GPX4 to inhibit ferroptosis. Nature.

[88] von Krusenstiern A.N., Robson R.N., Qian N., Qiu B., Hu F., Reznik E., Smith N., Zandkarimi F., Estes V.M., Dupont M. (2023). Identification of essential sites of lipid peroxidation in ferroptosis. Nat. Chem. Biol..

[89] Park E., Chung S.W. (2019). ROS-mediated autophagy increases intracellular iron levels and ferroptosis by ferritin and transferrin receptor regulation. Cell Death Dis..

[90] Jiang L., Kon N., Li T., Wang S.J., Su T., Hibshoosh H., Baer R., Gu W. (2015). Ferroptosis as a p53-mediated activity during tumour suppression. Nature.

[91] Niu B., Lei X., Xu Q., Ju Y., Xu D., Mao L., Li J., Zheng Y., Sun N., Zhang X. (2022). Protecting mitochondria via inhibiting VDAC1 oligomerization alleviates ferroptosis in acetaminophen-induced acute liver injury. Cell Biol. Toxicol..

[92] Wyatt J., Fernando S.M., Powell S.G., Hill C.J., Arshad I., Probert C., Ahmed S., Hapangama D.K. (2023). The role of iron in the pathogenesis of endometriosis: A systematic review. Hum. Reprod. Open.

[93] Van Langendonckt A., Casanas-Roux F., Donnez J. (2002). Iron overload in the peritoneal cavity of women with pelvic endometriosis. Fertil. Steril..

[94] Chen X., Zhou Y., Wu D., Shu C., Wu R., Li S., Huang Q., Shu J. (2021). Iron overload compromises preimplantation mouse embryo development. Reprod. Toxicol..

[95] Ni Z., Li Y., Song D., Ding J., Mei S., Sun S., Cheng W., Yu J., Zhou L., Kuang Y. (2022). Iron-overloaded follicular fluid increases the risk of endometriosis-related infertility by triggering granulosa cell ferroptosis and oocyte dysmaturity. Cell Death Dis..

[96] Wan Y., Gu C., Kong J., Sui J., Zuo L., Song Y., Chen J. (2022). Long noncoding RNA ADAMTS9-AS1 represses ferroptosis of endometrial stromal cells by regulating the miR-6516-5p/GPX4 axis in endometriosis. Sci. Rep..

[97] Beddows I., Fan H., Heinze K., Johnson B.K., Leonova A., Senz J., Djirackor S., Cho K.R., Pearce C.L., Huntsman D.G. (2023). Cell state of origin impacts development of distinct endometriosis-related ovarian carcinoma histotypes. Cancer Res..

[98] Atiya H.I., Frisbie L., Goldfeld E., Orellana T., Donnellan N., Modugno F., Calderon M., Watkins S., Zhang R., Elishaev E. (2022). Endometriosis-Associated Mesenchymal Stem Cells Support Ovarian Clear Cell Carcinoma through Iron Regulation. Cancer Res..

[99] Li Y., Zeng X., Lu D., Yin M., Shan M., Gao Y. (2021). Erastin induces ferroptosis via ferroportin-mediated iron accumulation in endometriosis. Hum. Reprod..

[100] Liang Z., Wu Q., Wang H., Tan J., Wang H., Gou Y., Cao Y., Li Z., Zhang Z. (2022). Silencing of lncRNA MALAT1 facilitates erastin-induced ferroptosis in endometriosis through miR-145-5p/MUC1 signaling. Cell Death Discov..

[101] Wang D., Tian Z., Zhang P., Zhen L., Meng Q., Sun B., Xu X., Jia T., Li S. (2023). The molecular mechanisms of cuproptosis and its relevance to cardiovascular disease. Biomed. Pharmacother..

[102] Lu J., Ling X., Sun Y., Liu L., Liu L., Wang X., Lu C., Ren C., Han X., Yu Z. (2023). FDX1 enhances endometriosis cell cuproptosis via G6PD-mediated redox homeostasis. Apoptosis.

[103] Zhou Y., Zhao X., Zhang L., Xia Q., Peng Y., Zhang H., Yan D., Yang Z., Li J. (2022). Iron overload inhibits cell proliferation and promotes autophagy via PARP1/SIRT1 signaling in endometriosis and adenomyosis. Toxicology.

[104] Li H., Yang H., Lu S., Wang X., Shi X., Mao P. (2023). Autophagy-dependent ferroptosis is involved in the development of endometriosis. Gynecol. Endocrinol..

[105] Chen K.W., Demarco B., Heilig R., Shkarina K., Boettcher A., Farady C.J., Pelczar P., Broz P. (2019). Extrinsic and intrinsic apoptosis activate pannexin-1 to drive NLRP3 inflammasome assembly. Embo J..

[106] Zhu P., Ke Z.R., Chen J.X., Li S.J., Ma T.L., Fan X.L. (2023). Advances in mechanism and regulation of PANoptosis: Prospects in disease treatment. Front. Immunol..

[107] Yan W.T., Zhao W.J., Hu X.M., Ban X.X., Ning W.Y., Wan H., Zhang Q., Xiong K. (2023). PANoptosis-like cell death in ischemia/reperfusion injury of retinal neurons. Neural Regen. Res..

[108] Place D.E., Lee S., Kanneganti T.D. (2021). PANoptosis in microbial infection. Curr. Opin. Microbiol..

[109] Zhou L., Cai E., Liu H., Cheng H., Ye X., Zhu H., Chang X. (2024). Extracellular ATP (eATP) inhibits the progression of endometriosis and enhances the immune function of macrophages. Biochim. Biophys. Acta Mol. Basis Dis..

[110] Park S., Lim W., Bazer F.W., Whang K.Y., Song G. (2019). Quercetin inhibits proliferation of endometriosis regulating cyclin D1 and its target microRNAs in vitro and in vivo. J. Nutr. Biochem..

[111] Soumoy L., Ghanem G.E., Saussez S., Journe F. (2022). Bufalin for an innovative therapeutic approach against cancer. Pharmacol. Res..

[112] Cho Y.J., Lee J.E., Park M.J., O’Malley B.W., Han S.J. (2018). Bufalin suppresses endometriosis progression by inducing pyroptosis and apoptosis. J. Endocrinol..

[113] Yi Z.H., Li S.Q., Ke J.Y., Wang Y., Zhao M.Z., Li J., Li M.Q., Zhu Z.L. (2022). Baicalein Relieves Ferroptosis-Mediated Phagocytosis Inhibition of Macrophages in Ovarian Endometriosis. Curr. Issues Mol. Biol..

[114] D’Amico R., Impellizzeri D., Cordaro M., Siracusa R., Interdonato L., Marino Y., Crupi R., Gugliandolo E., Macrì F., Di Paola D. (2022). Complex Interplay between Autophagy and Oxidative Stress in the Development of Endometriosis. Antioxidants.

[115] Song J., Ham J., Park S., Park S.J., Kim H.S., Song G., Lim W. (2023). Alpinumisoflavone Activates Disruption of Calcium Homeostasis, Mitochondria and Autophagosome to Suppress Development of Endometriosis. Antioxidants.

[116] Muzii L., Galati G., Di Tucci C., Di Feliciantonio M., Perniola G., Di Donato V., Benedetti Panici P., Vignali M. (2020). Medical treatment of ovarian endometriomas: A prospective evaluation of the effect of dienogest on ovarian reserve, cyst diameter, and associated pain. Gynecol. Endocrinol..

[117] Del Forno S., Mabrouk M., Arena A., Mattioli G., Giaquinto I., Paradisi R., Seracchioli R. (2019). Dienogest or Norethindrone acetate for the treatment of ovarian endometriomas: Can we avoid surgery?. Eur. J. Obs. Gynecol. Reprod. Biol..

[118] Choi J., Jo M., Lee E., Kim S.E., Lee D.Y., Choi D. (2022). Inhibition of the NLRP3 inflammasome by progesterone is attenuated by abnormal autophagy induction in endometriotic cyst stromal cells: Implications for endometriosis. Mol. Hum. Reprod..

[119] Choi J., Jo M., Lee E., Lee D.Y., Choi D. (2015). Dienogest enhances autophagy induction in endometriotic cells by impairing activation of AKT, ERK1/2, and mTOR. Fertil. Steril..

[120] Park S., Ham J., Yang C., Park W., Park H., An G., Song J., Hong T., Park S.J., Kim H.S. (2023). Melatonin inhibits endometriosis development by disrupting mitochondrial function and regulating tiRNAs. J. Pineal Res..

[121] Li Y., An M., Fu X., Meng X., Ma Y., Liu H., Li Q., Xu H., Chen J. (2023). Bushen Wenyang Huayu Decoction inhibits autophagy by regulating the SIRT1-FoXO-1 pathway in endometriosis rats. J. Ethnopharmacol..

[122] Zhao M., Zhang M., Yu Q., Fei W., Li T., Zhu L., Yao Y., Zheng C., Zhang X. (2022). Hyaluronic Acid-Modified Nanoplatforms as a Vector for Targeted Delivery of Autophagy-Related Gene to the Endometriotic Lesions in Mice. Front. Bioeng. Biotechnol..

[123] Gołąbek A., Kowalska K., Olejnik A. (2021). Polyphenols as a Diet Therapy Concept for Endometriosis-Current Opinion and Future Perspectives. Nutrients.

[124] Heard M.E., Melnyk S.B., Simmen F.A., Yang Y., Pabona J.M., Simmen R.C. (2016). High-Fat Diet Promotion of Endometriosis in an Immunocompetent Mouse Model is Associated With Altered Peripheral and Ectopic Lesion Redox and Inflammatory Status. Endocrinology.

[125] Ellis K., Wood R. (2023). The Comparative Invasiveness of Endometriotic Cell Lines to Breast and Endometrial Cancer Cell Lines. Biomolecules.

